# RiceMetaSys for salt and drought stress responsive genes in rice: a web interface for crop improvement

**DOI:** 10.1186/s12859-017-1846-y

**Published:** 2017-09-30

**Authors:** Maninder Sandhu, V. Sureshkumar, Chandra Prakash, Rekha Dixit, Amolkumar U. Solanke, Tilak Raj Sharma, Trilochan Mohapatra, Amitha Mithra S. V.

**Affiliations:** 1 ICAR-National Research Centre on Plant Biotechnology, LBS Building, Pusa Campus, New Delhi, 110012 India; 2 Shobhit University, Modipuram, Meerut, 250110 Uttar Pradesh India; 3 Department of Plant Molecular Biology and Bioinformatics, Tamil Nadu Agricultural University, Coimbatore, 641003 India; 4 Current address: Department of biotechnology, Keralverma faculty of science, Swami Vivekanand Subharti University, Meerut, 250005 Uttar Pradesh India; 5 Indian Council of Agricultural Research, Krishi Bhawan, New Delhi, 110001 India

**Keywords:** Rice, Meta-analysis, Salinity, Drought, DNA markers

## Abstract

**Background:**

Genome-wide microarray has enabled development of robust databases for functional genomics studies in rice. However, such databases do not directly cater to the needs of breeders. Here, we have attempted to develop a web interface which combines the information from functional genomic studies across different genetic backgrounds with DNA markers so that they can be readily deployed in crop improvement. In the current version of the database, we have included drought and salinity stress studies since these two are the major abiotic stresses in rice.

**Results:**

RiceMetaSys, a user-friendly and freely available web interface provides comprehensive information on salt responsive genes (SRGs) and drought responsive genes (DRGs) across genotypes, crop development stages and tissues, identified from multiple microarray datasets. ‘Physical position search’ is an attractive tool for those using QTL based approach for dissecting tolerance to salt and drought stress since it can provide the list of SRGs and DRGs in any physical interval. To identify robust candidate genes for use in crop improvement, the ‘common genes across varieties’ search tool is useful. Graphical visualization of expression profiles across genes and rice genotypes has been enabled to facilitate the user and to make the comparisons more impactful. Simple Sequence Repeat (SSR) search in the SRGs and DRGs is a valuable tool for fine mapping and marker assisted selection since it provides primers for survey of polymorphism. An external link to intron specific markers is also provided for this purpose. Bulk retrieval of data without any limit has been enabled in case of locus and SSR search.

**Conclusions:**

The aim of this database is to facilitate users with a simple and straight-forward search options for identification of robust candidate genes from among thousands of SRGs and DRGs so as to facilitate linking variation in expression profiles to variation in phenotype.

Database URL: http://14.139.229.201

**Electronic supplementary material:**

The online version of this article (10.1186/s12859-017-1846-y) contains supplementary material, which is available to authorized users.

## Background

Rice has the dual distinction of being a staple food crop for nearly 50% of world population and a genomic model crop for monocots which includes wheat and corn, the former being a staple cereal, and the latter a major source of animal nutrition [1]. In the last six decades, rice production has kept its growth in pace with the raising global food demand. However, rice production is supposed to further increase by 0.6 to 0.9% per year till 2050 to feed the additional 2 billion people expected to inhabit the earth by then [2–4]. Besides this major challenge of improving productivity, drought and salinity stress have emerged as the most important abiotic stresses that could endanger the sustainability of rice production. Since salinity and drought stress tolerance in rice are complex traits, in terms of their inheritance as well as molecular mechanism, researchers have been trying to address this problem by using genetic and genomic approaches [5–8].

One of the major approaches followed for dissecting complex traits such as drought and salt tolerance is the identification of QTLs by preliminary genetic mapping followed by fine mapping and identification of the candidate gene(s). Though this is a robust approach, it is laborious and time-consuming. With the advances in genomics, the entire process can be accelerated, especially, the steps after coarse mapping, even in crops not traditionally amenable for map-based cloning such as oil palm [9, 10]. In species where high-quality genome sequence information is available such as human, rice and Arabidopsis, microarray hybridization based genome-wide expression analysis is a very popular and useful technique to understand functional genomics [1, 11]. Expression microarray studies have been effectively used to characterize mutants and transgenic plants by comparing them with wild type [12–15]. Microarray generally identifies a large number of differentially expressed genes (DEGs) even in closely related individuals such as isogenic lines contrasting for a single trait [12]. Hence, one of the proven and effective ways to dissect complex traits is to combine genetic mapping with genome-wide transcriptome profiling of the parental genotypes which can help to narrow down the candidate gene(s) underlying the functional polymorphism in the QTL [13]. When huge numbers of genes from different biological materials are implicated in expression of a trait, meta-analysis provides a cost effective way to identify robust candidate gene(s) for trait improvement through breeding.

Meta-analysis aims at identification of statistically robust candidate genes from the already existing information such as the expression microarray data available in the public domain. In rice, using the microarray data, several publically accessible databases like OryzaExpress ([16], http://plantomics.mind.meiji.ac.jp/OryzaExpress/ ), RicePLEX ([17], http://www.plexdb.org/plex.php?database=Rice ), Rice Oligonucleotide Array database (ROAD) [18], RiceSRTFDB ([19], http://www.nipgr.res.in/RiceSRTFDB.html ), Oryzabase ([20], http://shigen.nig.ac.jp/rice/oryzabase/ ), QlicRice ([21], http://nabg.iasri.res.in:8080/qlic-rice ), OryGenesDB ([22], http://orygenesdb.cirad.fr/ ), RiceXPro ([23], http://ricexpro.dna.affrc.go.jp/) and qTeller ([24], http://qteller.com) and commercial platforms like Genevesigator and GeneMapper have been constructed. Of the freely available databases, ROAD is the most proficient and complete tool for meta-analysis of microarray data since it comprises of microarray data from multiple platforms, tissues, growth conditions and genotypes. Users can carry out gene expression analysis, co-expression and GO enrichment analysis and visualize the genes in a heat map. However, currently this database is not under maintenance and is not accessible. Orygene database is a functional genomic tool based on reverse genetics and hence offers flanking sequence tag (FST) based search. Oryzabase is a genome browser which provides information about rice development and anatomy of rice varieties, especially, wild varieties of rice. The qTeller database gives the list of genes in a QTL or a particular genomic interval whereas QlicRice lists the QTLs for various abiotic stresses, and different QTLs intervals. Though ROAD is a very useful forward functional genomic tool for identifying candidate genes for the trait of interest, for a plant breeder, ROAD is either not directly useful or very complex to use. On the other hand, qTeller and QlicRice are user-friendly but have not integrated the microarray data with QTL intervals. The commercially available tools such as Genevestigator are though highly informative, again intensive like ROAD and expensive to use.

To fine map the large QTL regions, plant breeders primarily look for polymorphisms between the parents of the mapping population in that defined region, in addition to the search for candidate genes using expression and bioinformatics approaches. Though SNPs are the most abundant and routinely used markers in vogue with low cost per data point [6], for investigating a well-defined genomic region in a cost-effective manner in a mapping population, the co-dominant and PCR-based microsatellites markers (also known as simple sequence repeats; SSRs) and intron length polymorphisms (ILP) or intron spanning markers (ISM) are more suitable. A database is readily available for searching ILP and ISM polymorphisms in any given gene but not SSRs [25].

Hence, we have constructed a database, named RiceMetaSys, especially intended for breeders, which directly combines the rice microarray data for salt and drought tolerance from both stress tolerant and susceptible genotypes along with their physical location and marker data. Since crop improvement researchers mainly concentrate on one trait at a time, we made the database trait specific. Though the focus is on salt and drought tolerance in the current version of RiceMetaSys, we intend to add more such important traits namely tolerance to leaf and panicle blast and high temperature. The purpose of microarray technology which is to enable biologists to study expression variation at a whole-genome level and link it to phenotypic variation [26] can be assisted by such efforts.

## Construction and content

### Data source

Microarray meta-analysis involves combining multiple independent but related microarray datasets into a meaningful context based profiles. Two or more experiments run on the same crop and treatment is not a sufficient enough justification for combining such datasets. Reproducibility and homogeneity of results across laboratories and datasets is also necessary, and in this context, Affymetrix platforms are considered more robust than other platforms [19]. Hence, the Affymetrix Microarray datasets comprising of 5 experiments (110 samples) pertaining to salinity and 6 experiments (131 samples) pertaining to drought treatment were retrieved from NCBI GEO database [27]. Expression data for salt stress was from nine varieties (Agami, M103, FL478, IR29, IR63731, Pokkali, CSR27, MI48 and IR64), representing vegetative and seedling growth stages and various sample tissues such as root, leaf and seedling (Additional file 1: Table S1). For drought stress, the expression datasets were from 10 different rice genotypes (Azucena, Bala, IRAT109, ZS97, IR64, Dhagaddeshi, IR20, Moroberekan, Nagina 22, and Nipponbare), representing eight different growth stages from seedling to panicle elongation, and seven different tissue samples covering vegetative to floral parts (Additional file 1: Table S1). The nature of the response of a genotype in terms of tolerance and sensitivity to a particular stress is also indicated in this table.

### Data processing and gene expression analysis

Since the treatment across experiments is not uniform, pre-processing (background correction and removal of batch effects) was carried out prior to gene expression analysis. Pre-processing of the microarray raw data from drought datasets was done using RMA (Robust Multi-Array Average) method and salinity datasets were normalized by log 2 transformation using R script from GEO2R. Non-experimental variation (batch effects owing to inter-laboratory and inter-batch differences) was removed using ComBat [28] tool in R. We have divided our data into drought and salt groups and removed batch effects separately. For each dataset, gene expression analysis was done using limma package v.3.28.21 and the R script from GEO2R with some slight modifications [29]. We have kept adjusted *p*-value 0.01 (for drought) 0.05 (for salt), Log FC value <−1 to 1>, and Average Expression >8 for both drought and salt microarray data sets.

### Database design

Affymetrix IDs of the salt and drought DEGs were converted to MSU7 IDs and RAP IDs by using OryzaExpress (http://bioinf.mind.meiji.ac.jp/OryzaExpress/ID_converter.php). A total of 1558 probe set IDs, either from salt responsive genes (SRGs) or drought responsive genes (DRGs) identified through analysis, did not have corresponding locus or gene IDs and hence were not considered for further processing. Physical positions and annotations were fetched from TIGR ([30], http://rice.plantbiology.msu.edu/). Microsatellites present in DRGs and SRGs were identified using BatchPrimer3 tool ([31], http://batchprimer3.bioinformatics.ucdavis.edu/cgi-bin/batchprimer3/batchprimer3.cgi) which not only identifies the microsatellites but also designs primers for the amplification of SSR fragments. The schematic representation of metadata analysis and RiceMetaSys design is given in Additional file 2: Figure S1. Server side scripting language used for RiceMetaSys was PHP with HTML5 in the front end and CSS with MySQL relational database at the backend. User interface framework employed was JQuery and JavaScript. Chart.js was used to generate graphs of expression profile of user-selected SRGs and DRGs in single or multiple rice genotypes. An external link option is provided in the SRG and DRG homepage to perform Gene Set Enrichment analysis (GSEA) and construct heat maps. Another external link enabled in the database is that of intron length based markers in rice. Database web server is XAMPP (Apache, MySQL, PHP, and Perl). The database is hosted in the server environment, FUJITSU PrimeRGY-Rx600S6 and Windows operating system. The database can be accessed at http://14.139.229.201.

## Utility and discussion

### Data statistics

**Fig. 1:**
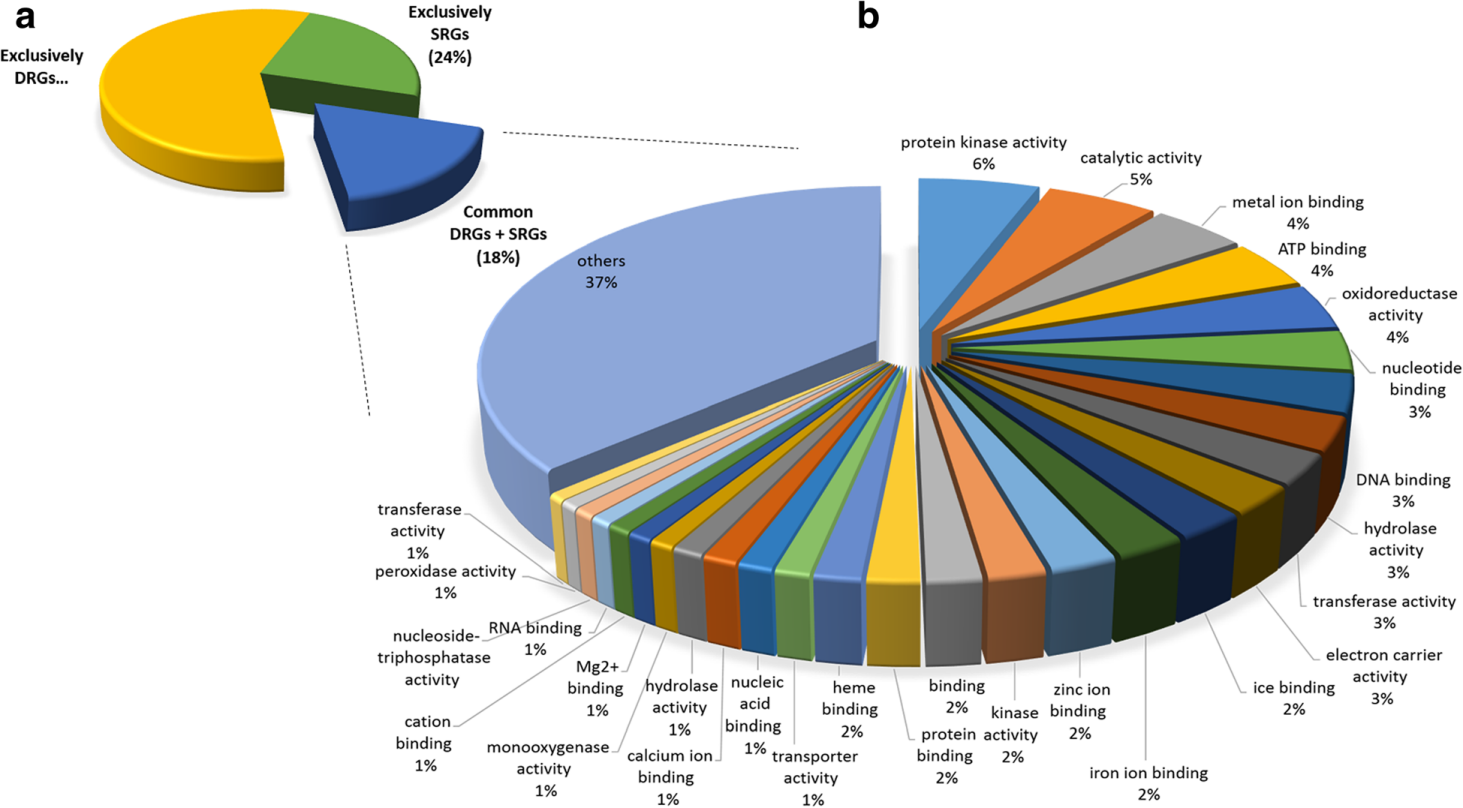
Distribution and functional annotation of overlapping SRGs and DRGs. (**a**) Distribution of the 12,501 DEGs present in the RiceMetaSys. 17% of the DEGs are common between DRGs and SRGs (**b**) Functional annotation of overlapping 2134 DEGs under salt and drought. These genes broadly regulate molecular processes belonging to protein phosphorylation, redox processes, electron carrier activity and DNA and RNA binding activities etc.

RiceMetaSys contains a total of 3120 salt responsive genes (SRGs) identified from salt microarray datasets and 9381 drought responsive genes (DRGs) from drought microarray datasets, after removing the duplicate entries (genes) identified across different studies within an abiotic stress group. Since both drought and salinity stresses induce osmotic stress in plants [32, 33], we searched for the genes common to both SRG and DRG datasets and found 2134 such genes (Fig. 1a). Interestingly, SRG set had only 986 (31.6%) unique salt specific genes, suggesting that imparting drought tolerance to plants would more often than not enhance their salinity tolerance too. GO ontology functional annotation of the 2134 common genes revealed that the maximum number of genes encoded undefined expressed proteins followed by zinc finger domain containing proteins and cytochromes (Fig. 1b). Thus the undefined expressed proteins encoding genes are a major class of candidate genes to target in combatting abiotic stress tolerance. For all the three groups namely SRG, DRG and genes commonly regulated in both the stresses, separate links (tabs) have been provided in the homepage of RiceMetaSys.

**Fig. 2:**
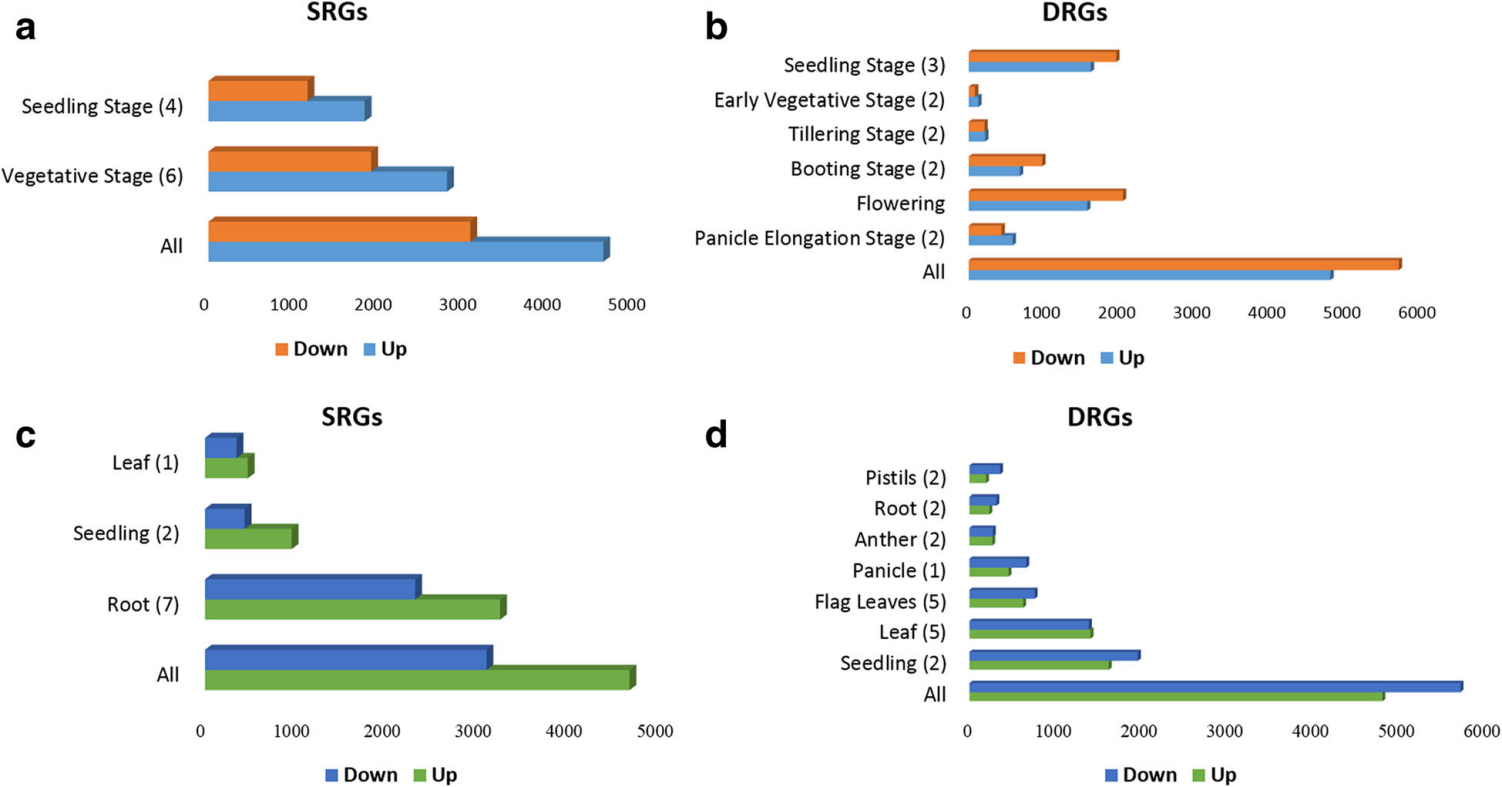
Distribution of DEGs in RiceMetaSys (**a**) and (**c**) Distribution of salt stress responsive genes across growth stages and tissues (**b**) and (**d**) Distribution of drought stress responsive genes across growth stages and tissues

The number of up and down regulated DEGs under salt and drought stress had a similar pattern i.e., the number of upregulated genes were more than downregulated genes (Fig. 2a). Based on the growth stage and tissue used in the experiments, the SRGs and DRGs were appropriately grouped. Comparison of DEGs among these groups revealed that this pattern was not true across the stages and tissues. The number of SRGs identified across tissues corresponded with the number of experiments conducted with a particular type of tissue (Fig. 2b). For instance, in salt microarray experiments, the root was the most often used tissue (7 times) and hence the number of SRGs from this tissue was more (Fig. 2c). Similarly, in DRGs, the DEGs were more in leaves collected at vegetative stage and entire seedling assays as the former was the most frequently sampled tissue (8 times) and the latter had the entire plant (Fig. 2d). Under drought, at flowering stage and in flag leaf and anther tissues, proportion of downregulated genes was slightly higher (53.65%, 52.75% and 63.15%; Fig. 2b and d). Similarly, under salinity, leaves had higher proportion of down regulated genes (65.5%; Fig. 2c). Comparison of DRGs in reproductive tissues revealed that the up and down regulated DEGs were nearly equal in pistils (51.3% and 48.7%) while in anthers, the number of down-regulated genes was nearly twice that of up regulated ones (63.15% and 36.85%).

**Fig. 3:**
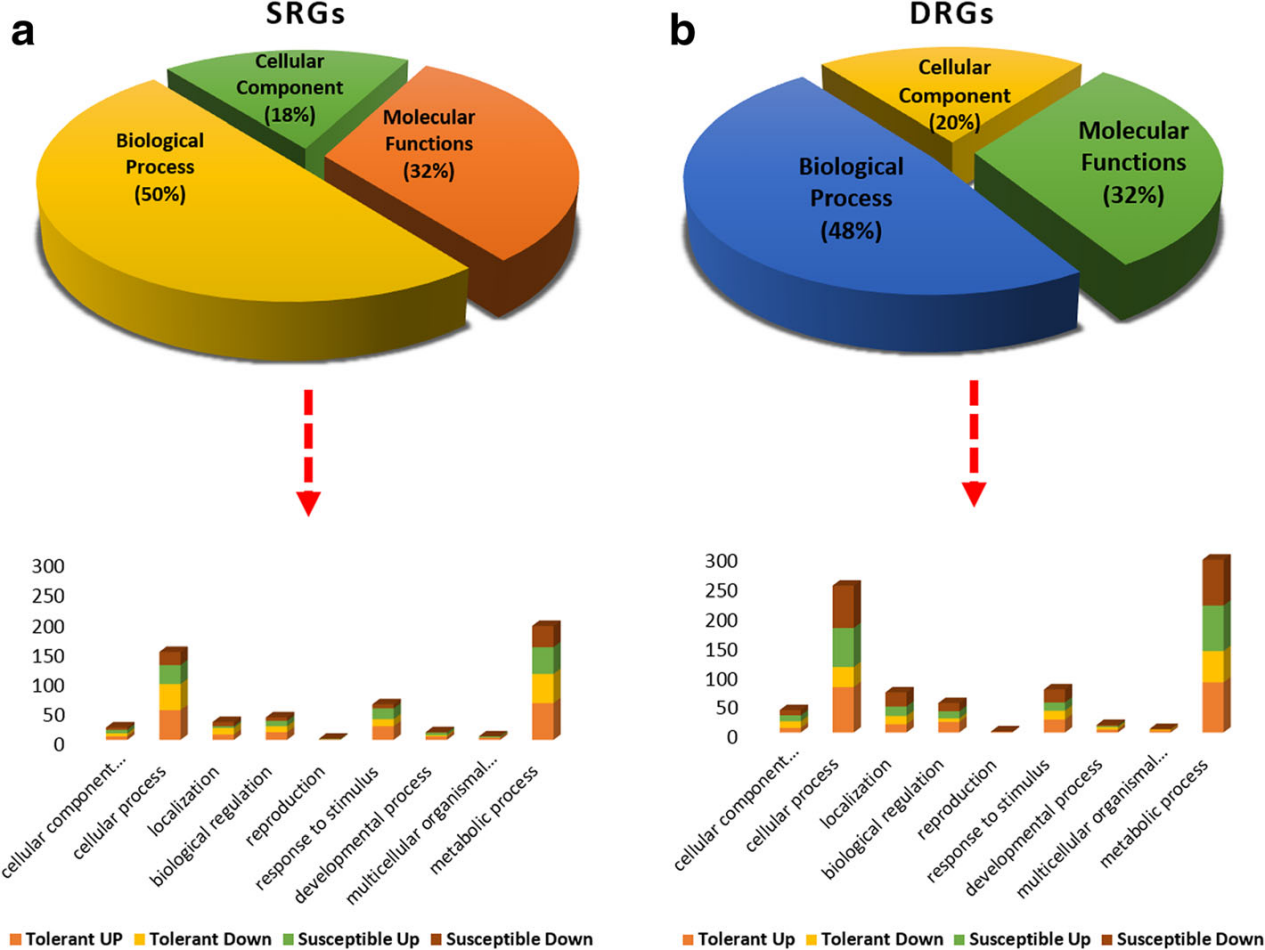
Gene Ontology of the identified stress responsive genes (**a**) Majority of the identified SRGs corresponds to biological process (45.17%) followed by molecular function (31%) (**b**) The distribution pattern was vice-versa for DRGs with major proportion of the identified genes in the category molecular function (46.2%) followed by biological process (29.4%)

GO annotation of the SRGs and DRGs revealed almost similar proportion of genes under cellular components and pathways. However, under molecular functions, and biological processes the abundance was more in the former than the latter for DRGs (46.22% and 29.42%) and vice-versa for SRGs (31.01% and 45.17%) (Fig. 3a and b). Comparison of known salt tolerant and susceptible genotypes (Additional file 1: Table S1, and Fig. 3a and b) revealed that more SRGs were from salt tolerant genotypes (143) than susceptible genotypes (116). In the case of DRGs, the trend was reverse with more number of DRGs found in drought sensitive genotypes (621 against 567). While under drought the number of up and down regulated across tolerant and susceptible genotypes was comparable, in salinity the number of upregulated genes were more in salt tolerant genotypes than all the other three classes. Under metabolic processes, the number of upregulated SRGs in tolerant genotypes was the highest (Fig. 3a). Under cellular processes, the number of downregulated DRGs in susceptible genotypes was the highest (Fig. 3b).

**Fig. 4:**
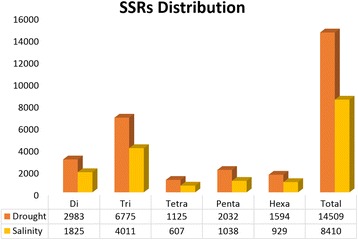
Distribution of microsatellites in the DRGs and SRGs of rice

A total of 12,070 SSRs were found in DRGs (8451) and SRGs (3619) meeting the following parameters set for their mining: dinucleotide units repeated at least 6 times, trinucleotide motifs 5 times, tetranucleotide repeats 5 times, pentanucleotides repeats 3 times and hexanucleotide repeats 3 times. Trinucleotide motifs were the most abundant in both DRGs (51%) and SRGs (50%) as already reported in rice [34]. However, dinucleotide repeats in SRGs and DRGs were much lower (Fig. 4; 21.1% and 20.7%) as compared to previous reports [34, 35]. Tetranucleotides were the least abundant in both DRGs (2.5%) and SRGs (2.6%). Nearly, one-fifth (24.6%) of the repeats were class I microsatellites.

### Database features vis-à-vis available datasets

**Fig. 5:**
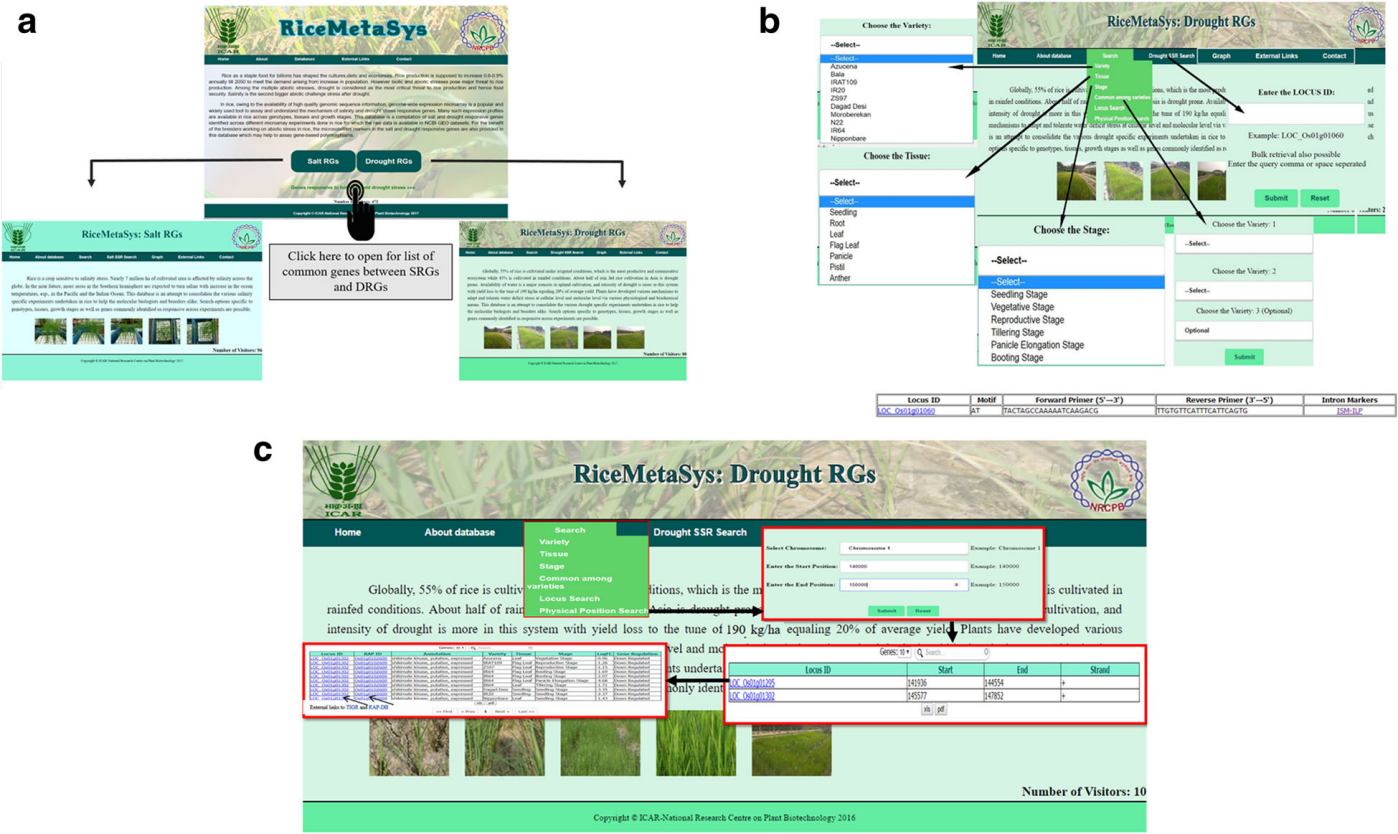
An overview of RiceMetaSys (**a**) Snapshot of the RiceMetaSys database showing the homepage with links to SRGs, DRGs and common genes between SRGs and DRGs. (**b**) Search options such as variety, tissue, stage, commonly expressed genes among varieties and SSRs. (**c**) Physical position search option and its output. Selecting the ‘Physical position” search opens a window in which chromosome number and the genomic interval (start and end point) are to be provided as input by the user. This lists the stress responsive genes in the interval in another window. Selecting individual genes from this list provides detailed information on its stress responsiveness

More often than not, researchers focus on a specific trait and aim to understand the molecular mechanisms governing that trait. Further, crosstalk at the molecular level is extremely well known across stress responses [36, 37]. Hence, besides separate links for SRGs and DRGs, another link for genes common to SRG and DRG has been provided in the home page of RiceMetaSys (Fig. 5a). Biologically, it is well known that the response to any stress is genotype, stage and tissue specific. For instance, the well-known salt tolerant QTL in chromosome 1 (Saltol) of rice confers tolerance only at the vegetative stage but not at reproductive stage [7]. Hence, along with genotype specific search, both growth stage and tissues specific searches were enabled in our RiceMetaSys database in all the three links (Fig. 5b). Any desired stages/tissue/variety can easily be selected from the drop down menu by the user under appropriate search option. The output gives a list of stress responsive genes with their gene IDs (LOC_ID), annotation, log fold change (FC) and the direction of regulation (up or down) specific to the search option (Fig. 5b). Data can be sorted according to FC values or direction of regulation of DEGs by clicking on each heading as per user’s requirement. To enable this, the output format has been kept simple and in text format with limited graphics. Another important feature enabled in the RiceMetaSys web interface is the nature of output from stage and tissue specific search: rather than just a list of DEGs, complete information on the gene across genotypes is given with other details so that the importance of the gene can be easily deciphered (Fig. 5b). Visualization of output in multiples of 10 genes from 10 to 50 of genes (SRG/DRG) has also been enabled. In addition, the user has the choice of downloading the results in MS-Excel and PDF format.

From the breeders’ and farmers’ perspective, the stress incidence at the reproductive stage is more important than that at vegetative stage since the former affects both economic yield and quality of the produce more severely. Interestingly, from the available data, it was apparent that there were no microarray datasets available from reproductive stage or tissues for salt stress whereas in drought four of the six experiments analyzed had data from reproductive tissues or stage. Of late, QTLs for reproductive stage salinity stress tolerance have been mapped in rice [6, 7]. Thus, generating genome-wide expression data at reproductive stage would be very useful for fine mapping of the QTLs identified in those studies.

A comparative analysis of the available databases along with RiceMetaSys has been carried out based on multiple parameters such as general features, expression type, co-expression analysis, trait specificity, and marker type and output format (Table 1). ROAD database is the best tool available for expression analysis and covers most of the microarray experiments for salinity and drought. However, RiceMetaSys has more microarray experiment datasets (Affymetrix) for salinity and drought as ROAD database has not been updated since 2012 and is currently unavailable. Although ROAD database includes all biotic and abiotic traits for rice, expression analysis can be done with only one experiment at a time. Consequently, the meta-analysis in ROAD is not trait specific. The same issue exists with RicePLEX database as well. We have not enabled co-expression, pathway analysis and protein-protein interactions in our database because we wanted to keep it simple and user-friendly for the breeders. Still, an external link has been provided for Gene Set Enrichment analysis (GSEA) and construction of heat maps. Results (output of gene IDs) obtained from search performed with our database can be directly given as input to GSEA.

**Table 1:** Comparison of main features of different rice expression databases

Parameter	RiceMetaSys	ROAD*	Rice SRTFdb	Rice-Plex	RiceXpro	Qteller	QlicRice
Tissue/stage/genotype specific expression	Yes	Yes	No	No (individual datasets)	Yes	Yes (need to select experiment)	No
Co-expression analysis	No(external link provided)	Yes	No	No	No	No	No
Trait specific search	Yes	Yes (but meta-analysis is not trait specific)	Yes	Yes	No	No	Yes (For QTLs)
Output format	Table and graphs	Heatmap, table and graphs	Table	Heatmap, table and graphs	Map chart and table	Table	Table
Marker information	SSRs, ISM-ILP	No	No	No	No	No	No
Bulk Acceptance/Retrieval	Yes	Yes	Yes	Yes	Yes	Retrieval possible but not acceptance	Yes
Other Details	Various search options for better comparison; Genes common between traits as well as among varieties can be retrieved; DEGs between two markers can be retrieved	Single and multiple platform probe search; Meta profiling possible	Focus on TFs; Common genes between traits can be retrieved	Based on rice and 15 other plant species; Homology among various species	Genes can be viewed from field/development and plant hormone microarray datasets	Based on expression studies in major crop species; Genes between two physical coordinates can be retrieved	QTL specific database; Genes in the QTL interval can be retrieved

*Currently not available

### Common genes, locus and physical position search

Molecular mechanisms that impart tolerance to any abiotic stress can be either universal or genotype specific. The possibility of allelic diversity, epistasis and GXE interactions complicate the expression profile further. Thus, the robust candidate genes for tolerance could be the ones that have a similar pattern of expression in tolerant genotypes as against sensitive genotypes. Hence, comparison of SRGs and DRGs, up to three genotypes, has been enabled in RiceMetaSys which gives the list of commonly regulated genes across the genotypes selected (Fig. 5b). This search provision is also useful for short-listing of genes for their functional characterization. The ‘common genes search across varieties’ is a unique feature of RiceMetaSys.

For a researcher interested in a specific gene, for its plausible role in imparting salinity or drought stress tolerance, the ‘Locus search’ option is a convenient tool (Fig. 5b). The LOC IDs have been hyperlinked with the genome browser for access to more information. Bulk retrieval of data is also possible in ‘Locus search’ without any limit on number of genes but per page view is restricted to a maximum of 50 genes for the sake of clarity.

**Fig. 6:**
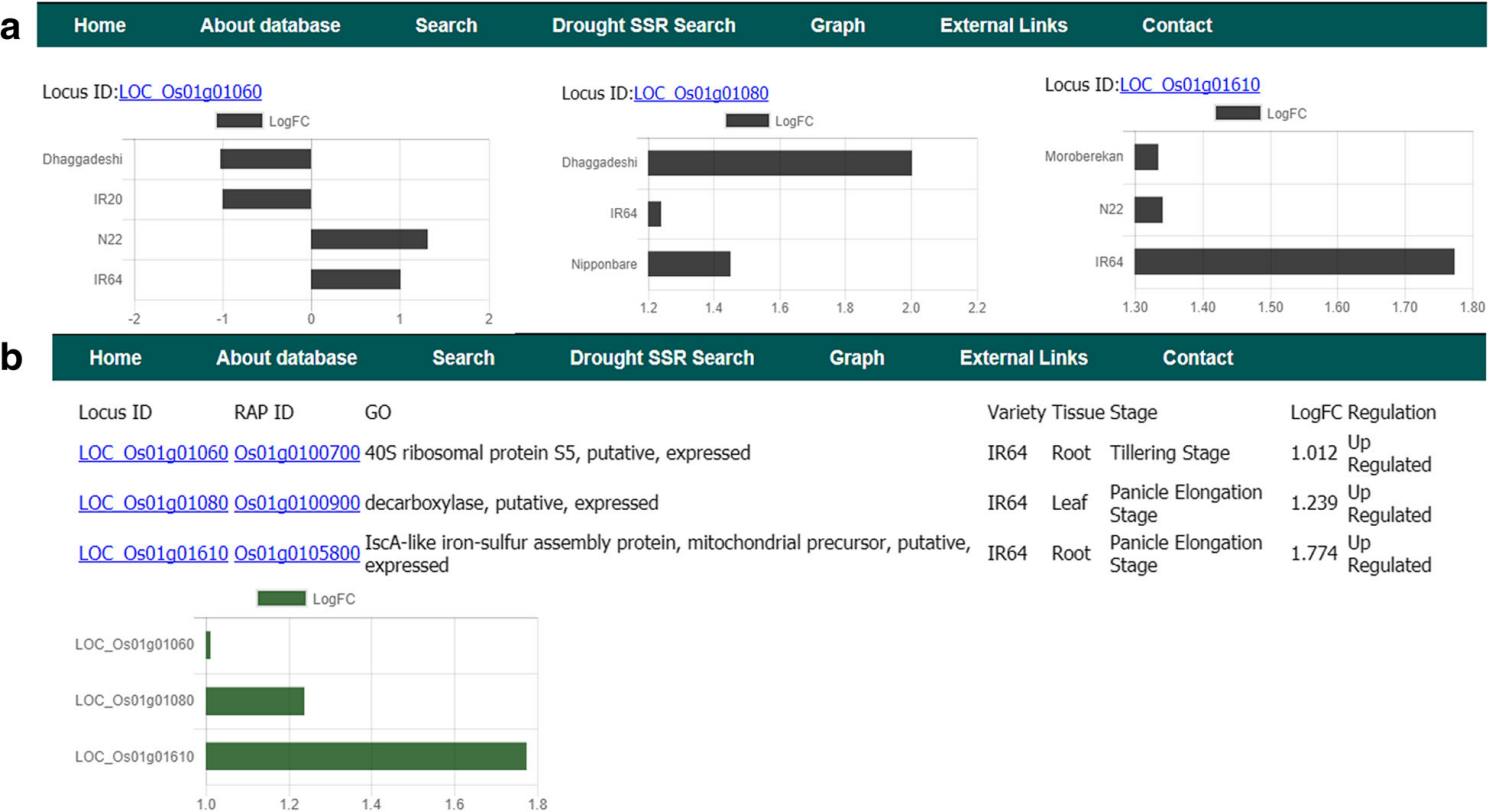
Snapshot of Graph tool in RiceMetaSys. User can submit up to 10 locus ID’s and can view expression profile of, (**a**) candidate genes among different varieties (shown in black bars) or, (**b**) candidate genes within a variety e.g. Dhaggadeshi (shown in green bars). *for the sake of clarity we have shown data of 3 genes (locus IDs)

For the analysis of genes present in the known and novel QTLs, it would be very useful if the stress responsive genes present in a given genomic interval are known. This would help in both fine mapping and gene validation (to pick the right candidate). RiceMetaSys makes this possible with the ‘physical position search’ tool (Fig. 5c). The workflow for using this option is explained in Additional file 3: Figure S2. Graphical representation of expression profiles of selected candidate genes, up to 10, in a single or multiple genotypes is also available in the database. The input required for this option is a list of locus IDs. This is a very useful tool to check whether a given candidate gene is functioning in a universal or variety specific manner (Fig. 6).

Once the list of stress responsive genes is available, the next logical and immediate step is to look for locus specific DNA markers in that interval so as to test for polymorphisms in the parents of the QTL mapping population for those markers. Though SNPs are the makers of choice [38, 39], fine mapping programs prefer simple-to-genotype markers that are also amenable to large scale genotyping. Both SSRs and Intron length spanning markers or intron length polymorphisms (ISM-ILP) fit this description perfectly [25, 40]. Hence, a separate tab for SSR search has been provided in the database. By submitting the list of LOC IDs found in a given physical interval, SSRs present, if any, in the genes would be displayed along with the SSR motif and primer information so that the polymorphisms can be surveyed by the researcher (Fig. 5c). If the researcher wants to look for ISM-ILP polymorphism in the SRGs or DRGs, an external link to ISM-ILP database (http://webapp.cabgrid.res.in/ismdb/database.html) has been provided with each LOC ID, under the SSR search tab. The marker polymorphisms identified can also be directly used for marker assisted selection in both back cross and recombinant breeding programs.

### RiceMetaSys: Utility for rice breeders

Universal and robust candidate genes are preferred by breeders for exploitation in crop improvement. Using ‘common variety search’ tool and graphics tab for visualization of expression profile across varieties, it is possible for breeders to select the robust candidates (Fig. 5b). Further, they can select the DEGs in the known major QTL intervals by using the ‘physical position search’ option (Fig. 5C and Additional file 3: Figure S2). If desired, visualization of expression profile of DEGs in QTL intervals can also be done. Since growth stage specific tolerance is established in rice for both drought and rice, breeders might be interested in stage specific DEG option enabled in the database. For precise breeding applications, breeders can use the SSR and ISM-ILP polymorphism links and straightaway use the primers as PCR based markers (Fig. 5c). Since the database is simple in construction, breeders can use it intuitively without any guidance.

## Conclusions

Meta-analysis of multiple microarray datasets provides a means for identification of robust candidate genes for the trait of interest. RiceMetaSys is a user-friendly web interface mainly intended for rice breeders for identification of salt and drought responsive genes in QTL intervals and those common to multiple stages, tissues and genetic backgrounds in rice. The SSR and ISM-ILP marker information provided is expected to help the molecular geneticists and breeders alike in their breeding and fine mapping efforts. Our purpose of developing RiceMetaSys is to provide a separate link for each and every economically important biotic and abiotic stress in rice. In the current version, we have accomplished it for salt and drought tolerance. In the next, we would be adding more important traits like extreme temperature tolerance and leaf and panicle blast resistance. We will be integrating the RNA-seq data for these traits as well in the future.

## Additional files

Additional file 1: Table S1. Detailed information about the microarray datasets retrieved from NCBI GEO database. (DOCX 17 kb)

Additional file 2: Figure S1. Schematic diagram of the RiceMetaSys database. Datasets were downloaded from the NCBI GEO and then were analyzed using GEO2R based script for the identification of DEGs. A comprehensive web based interface was developed to provide useful search information related to DEGs like commonly expressed genes, common genes across genotypes and DEGs in given physical intervals and genic microsatellites. (PPTX 610 kb)

Additional file 3: Figure S2. Detailed workflow for Physical position search (DOCX 36 kb)

## Abbreviations

DEG: Differentially expressed genes
DRG: Drought responsive genes
ILP: Intron length polymorphism
ISM: Intron spanning markers
QTL: Quantitative trait loci
SRG: Salt responsive genes
