# Supplementary material for: RiceMetaSys for salt and drought stress responsive genes in rice: a web interface for crop improvement
Source: BMC Bioinformatics. 2017 Sep 30;18:432. doi: 10.1186/s12859-017-1846-y (PMC5622590; doi:10.1186/s12859-017-1846-y)
Supplement: Supplementary file 1 — Detailed information about the microarray datasets retrieved from NCBI GEO database. (DOCX 17 kb) [file 12859_2017_1846_MOESM1_ESM.docx]

**Additional file 1: Table S1:** Details of the microarray datasets used for developing the database RiceMetaSys

| **Salinity** | | | | | | |
| --- | --- | --- | --- | --- | --- | --- |
| **S.No** | **GEO Accession ID** | **No.of Samples** | **Rice Genotype** | **Growth Stage** | **Tissue** | **Response to Salt Stress** |
| 1 | GSE4438 | 24 | Agami | Vegetative Stage | Root | Tolerant |
|  |  |  | M103 | Vegetative Stage | Root | Susceptible |
| 2 | GSE13735 | 11 | FL478 | Vegetative Stage | Root | Tolerant |
|  |  |  | IR29 | Vegetative Stage | Root | Susceptible |
| 3 | GSE14403 | 23 | IR63731 | Vegetative Stage | Root | Tolerant |
|  |  |  | Pokkali | Vegetative Stage | Root | Tolerant |
| 4 | GSE16108 | 16 | CSR27 | Seedling Stage | Seedling | Tolerant |
|  |  |  | MI48 | Seedling Stage | Seedling | Susceptible |
| 5 | GSE58603 | 36 | IR64 | Seedling Stage | Leaf | Susceptible |
|  |  |  | IR64 | Seedling Stage | Root | Susceptible |

| **Drought:** | | | | | | |
| --- | --- | --- | --- | --- | --- | --- |
| **S.No** | **GEO Accession ID** | **No.of Samples** | **Rice Genotype** | **Growth Stage** | **Tissue** | **Response to Drought Stress** |
| 1 | GSE24048 | 12 | Azucena | Vegetative Stage | Leaf | Tolerant |
|  |  |  | Bala | Vegetative Stage | Leaf | Tolerant |
| 2 | GSE25176 | 16 | IRAT109 | Reproductive Stage | Flag Leaf | Tolerant |
|  |  |  | ZS97 | Reproductive Stage | Flag Leaf | Susceptible |
| 3 | GSE26280 | 36 | IR64 | Booting Stage | Flag Leaf | Susceptible |
|  |  |  | IR64 | Booting Stage | Panicle | Susceptible |
|  |  |  | IR64 | Panicle Elongation Stage | Flag Leaf | Susceptible |
|  |  |  | IR64 | Panicle Elongation Stage | Root | Susceptible |
|  |  |  | IR64 | Tillering Stage | Leaf | Susceptible |
|  |  |  | IR64 | Tillering Stage | Root | Susceptible |
| 4 | GSE41647 | 18 | Dagad Desi | Seedling Stage | Seedling | Tolerant |
|  |  |  | IR20 | Seedling Stage | Seedling | Susceptible |
| 5 | GSE57154 | 35 | Moroberekan | Reproductive Stage | Anther | Tolerant |
|  |  |  | Moroberekan | Reproductive Stage | Pistils | Tolerant |
|  |  |  | N22 | Reproductive Stage | Anther | Tolerant |
|  |  |  | N22 | Reproductive Stage | Pistils | Tolerant |
| 6 | GSE81253 | 14 | Nipponbare | Seedling Stage | Leaf | Tolerant |
|  |  |  | Nipponbare | Vegetative Stage | Leaf | Tolerant |
|  |  |  | Nipponbare | Reproductive Stage | Flag Leaf | Tolerant |
