# Supplementary material for: RiceMetaSys for salt and drought stress responsive genes in rice: a web interface for crop improvement
Source: BMC Bioinformatics. 2017 Sep 30;18:432. doi: 10.1186/s12859-017-1846-y (PMC5622590; doi:10.1186/s12859-017-1846-y)
Supplement: Supplementary file 2 — Schematic diagram of the RiceMetaSys database. Datasets were downloaded from the NCBI GEO and then were analyzed using GEO2R based script for the identification of DEGs. A comprehensive web based interface was developed to provide useful search information related to DEGs like commonly expressed genes, common genes across genotypes and DEGs in given physical intervals and genic microsatellites. (PPTX 610 kb) [file 12859_2017_1846_MOESM2_ESM.pptx]

## Slide 1
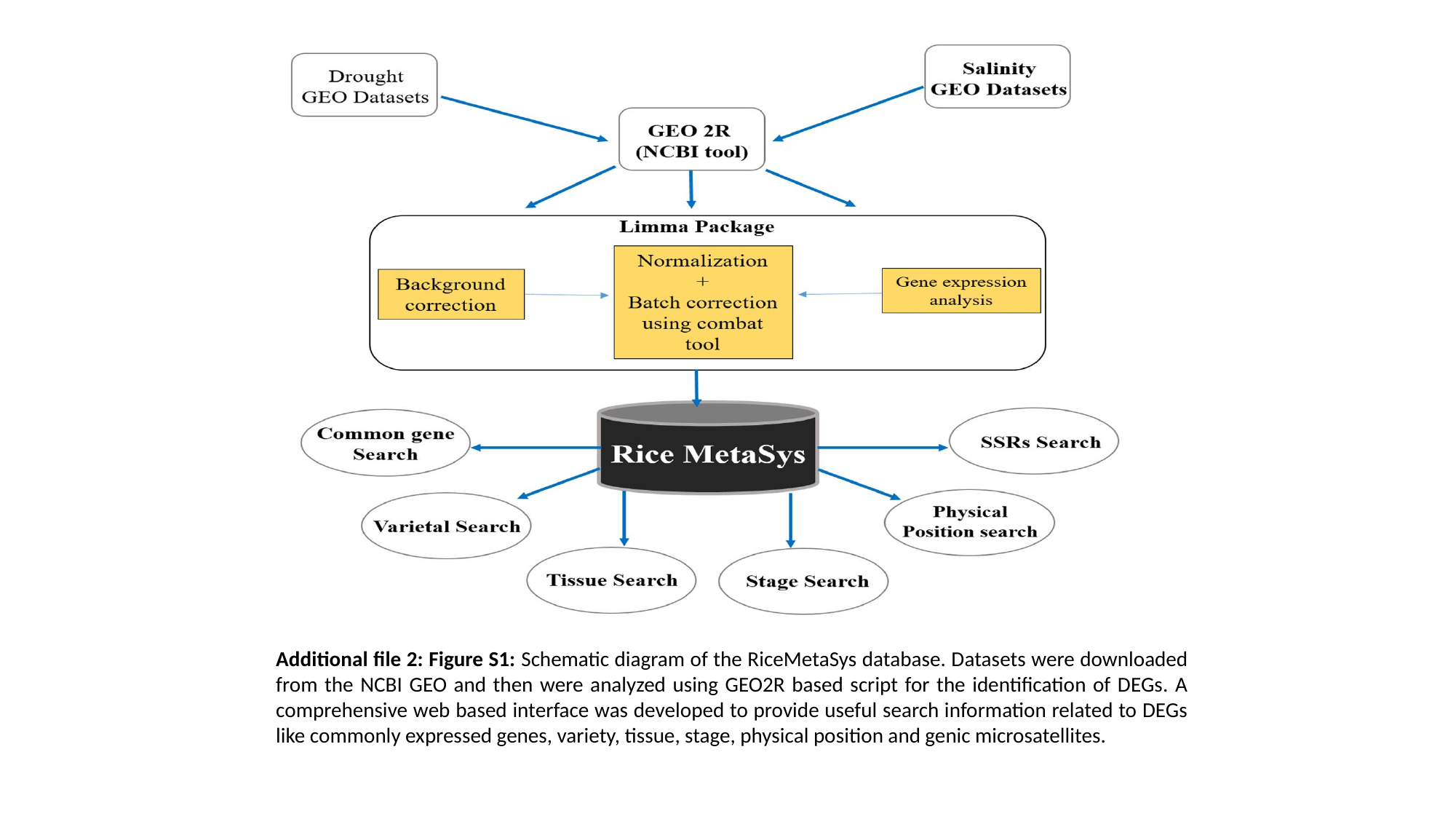

Additional file 2: Figure S1: Schematic diagram of the RiceMetaSys database. Datasets were downloaded from the NCBI GEO and then were analyzed using GEO2R based script for the identification of DEGs. A comprehensive web based interface was developed to provide useful search information related to DEGs like commonly expressed genes, variety, tissue, stage, physical position and genic microsatellites.
