# Supplementary material for: RiceMetaSys for salt and drought stress responsive genes in rice: a web interface for crop improvement
Source: BMC Bioinformatics. 2017 Sep 30;18:432. doi: 10.1186/s12859-017-1846-y (PMC5622590; doi:10.1186/s12859-017-1846-y)
Supplement: Supplementary file 3 — Detailed workflow for Physical position search (DOCX 36 kb) [file 12859_2017_1846_MOESM3_ESM.docx]

**Additional file 3:** **Figure S2:** Detailed workflow for ‘Physical position search’

**Physical Position Search**

**Input value**

| **Chromosome Number** | Chromosome 1 |
| --- | --- |
| **Start Position** | 140000 |
| **End Position** | 150000 |

| **Locus** | **Start** | **End** | **Strand** |
| --- | --- | --- | --- |
| LOC_Os01g01295 | 145577 | 147852 | **+** |
| LOC_Os01g01302 | 141936 | 144554 | **+** |

| **Locus ID** | **RAP ID** | **Annotation** | **Variety** | **Tissue** | **Stage** | **LogFC** | **Gene Regulation** |
| --- | --- | --- | --- | --- | --- | --- | --- |
| LOC_Os01g01302 | Os01g0102600 | shikimate kinase, putative, expressed | Azucena | Leaf | Vegetative Stage | -0.96 | Down Regulated |
| LOC_Os01g01302 | Os01g0102600 | shikimate kinase, putative, expressed | IRAT109 | Flag Leaf | Reproductive Stage | -1.26 | Down Regulated |
| LOC_Os01g01302 | Os01g0102600 | shikimate kinase, putative, expressed | ZS97 | Flag Leaf | Reproductive Stage | -1.15 | Down Regulated |
| [LOC_Os01g01302](http://rice.plantbiology.msu.edu/cgi-bin/ORF_infopage.cgi?orf=LOC_Os01g01302) | Os01g0102600 | shikimate kinase, putative, expressed | IR64 | Flag Leaf | Booting Stage | -1.69 | Down Regulated |
| [LOC_Os01g01302](http://rice.plantbiology.msu.edu/cgi-bin/ORF_infopage.cgi?orf=LOC_Os01g01302) | Os01g0102600 | shikimate kinase, putative, expressed | IR64 | Flag Leaf | Booting Stage | -2.07 | Down Regulated |
| LOC_Os01g01302 | Os01g0102600 | shikimate kinase, putative, expressed | IR64 | Flag Leaf | Panicle Elongation Stage | -4.68 | Down Regulated |
| LOC_Os01g01302 | Os01g0102600 | shikimate kinase, putative, expressed | IR64 | Leaf | Tillering Stage | -1.71 | Down Regulated |
| LOC_Os01g01302 | Os01g0102600 | shikimate kinase, putative, expressed | Dagad Desi | Seedling | Seedling Stage | -3.35 | Down Regulated |
| LOC_Os01g01302 | Os01g0102600 | shikimate kinase, putative, expressed | IR20 | Seedling | Seedling Stage | -2.37 | Down Regulated |
| LOC_Os01g01302 | Os01g0102600 | shikimate kinase, putative, expressed | Nipponbare | Leaf | Seedling Stage | -1.43 | Down Regulated |
| LOC_Os01g01302 | Os01g0102600 | shikimate kinase, putative, expressed | Nipponbare | Leaf | Vegetative Stage | -3.35 | Down Regulated |

**Genome Browser**
